# Time Trend of Upper Gastrointestinal Cancer Incidence in China from 1990 to 2019 and Analysis Using an Age–Period–Cohort Model

**DOI:** 10.3390/curroncol29100588

**Published:** 2022-10-06

**Authors:** Yongtian Lin, Zengqing Guo, Shuna Huang, Jingyu Ma, Zhisheng Xiang, Yongying Huang, Yan Zhou, Wanqing Chen

**Affiliations:** 1 Department of Epidemiology, Clinical Oncology School of Fujian Medical University, Fujian Cancer Hospital, Fuzhou 350014, China; 2 Department of Medical Oncology, Clinical Oncology School of Fujian Medical University, Fujian Cancer Hospital, Fuzhou 350014, China; 3 Department of Clinical Research and Translation Center, The First Affiliated Hospital of Fujian Medical University, Fuzhou 350004, China; 4 National Cancer Center, National Clinical Research Center for Cancer, Cancer Hospital Chinese Academy of Medical Sciences, Beijing 100021, China

**Keywords:** upper gastrointestinal cancer, incidence trend, age–period–cohort model, Joinpoint

## Abstract

The aim of this study was to investigate the upper gastrointestinal cancer incidence trend in China from 1990 to 2019 with Joinpoint software and to evaluate the age effect, cohort effect, and period effect using the age–period–cohort model, with the data obtained from the Global Burden of Disease, Injuries, and Risk Factors Study. The crude incidence rate (CR) of upper gastrointestinal cancer in China increased from 41.48/100,000 in 1990 to 62.64/100,000 in 2019, and the average annual percent change (AAPC) was 1.42 (*p* < 0.05). The age-standardized incidence rate (ASIR) decreased from 50.77/100,000 to 37.42/100,000, and the AAPC was −1.12 (*p* < 0.05). The net drift was −0.83 *(p* < 0.05), and the local drifts in the 35–79 age groups of males and all age groups of females were less than 0 *(p* < 0.05). The age effect showed that the upper gastrointestinal cancer onset risk gradually increased with age, the period effect was fundamentally manifested as a downward trend in onset risk after 2000, and the cohort effect indicated the decreased onset risk of the overall birth cohort after 1926. The ASIR of upper gastrointestinal cancer in China from 1990 to 2019 showed a downward trend, and the onset risk indicated the age, period, and cohort effects.

## 1. Introduction

In 2020, the worldwide incidences of gastric cancer and esophageal cancer were 11.3/100,000 and 6.3/100,000, yielding 1,089,103 and 604,127 new cases, respectively, with three-quarters of them occurring in developing countries [1]. As the largest developing country in the world, China ranks fourth and third in the world in the incidences of gastric cancer (20.6/100,000) and esophageal cancer (13.8/100,000), respectively [2]. Due to its large population base, China has the largest number of patients with gastric cancer and esophageal cancer in the world, accounting for 53.70% and 43.94%, respectively, of patients worldwide [1]. China is among the few countries suffering a high incidence of both esophageal cancer and gastric cancer; therefore, active measures must be taken to effectively prevent these cancers.

Given the high incidence of esophageal cancer and gastric cancer, China initiated an upper gastrointestinal cancer screening program in the high-incidence rural areas in 2005. Both the esophagus and stomach are examined in a single endoscopic screening for early detection of esophageal cancer and gastric cancer. This program remains ongoing and has gradually covered most provinces. Both the Cancer Early Diagnosis Program and the Early Treatment Program in the Huai River Basin initiated in 2009 and the Cancer Early Diagnosis and Early Treatment Program in Urban China initiated in 2012 included upper gastrointestinal cancer in their screening. Initiation of the national endoscopic screening program may have a significant impact on reducing China’s incidence of gastric cancer [3]. A study on the ten-year follow-up of six rural areas with a high incidence of upper gastrointestinal cancer revealed that the incidences of esophageal cancer and gastric cancer can be reduced by 20% and 14%, respectively, simply by screening one-third of the residents in the high-risk regions, indicating that endoscopic screening is an effective way to prevent upper gastrointestinal cancer [4].

Since esophageal cancer is different from gastric cancer in epidemiological characteristics and pathogenesis [5,6], many previous studies analyzed the prevalence of esophageal cancer and gastric cancer separately. However, both cancers show a high incidence in China, and large-scale combined upper gastrointestinal cancer screening has been performed specifically. Esophageal cancer is not exactly the same as gastric cancer in risk factors, but they indeed share some common risk factors. Epidemiological analysis of esophageal cancer and gastric cancer together as upper gastrointestinal cancer can better reflect the high-prevalence situation of esophageal cancer and gastric cancer in China and can better suit China’s initiative in cancer prevention and control.

In this study, we analyzed the incidence trend of upper gastrointestinal cancer and evaluated the influence of age, period, and birth cohort on the onset risk of upper gastrointestinal cancer. This study is helpful for China to develop more effective upper gastrointestinal cancer prevention strategies to reduce the upper gastrointestinal disease burden in China, and even in the world, and provides a scientific basis for the future evaluation of the screening effect.

## 2. Methods

### 2.1. Data Source

All data in this study were obtained from the Global Burden of Disease, Injuries, and Risk Factors Study (GBD) [7]. The GBD program aims to assess the disease burden of multiple diseases, harms, and risk factors around the world and regularly publishes the data of incidence, mortality, and disability-adjusted life years by country, year, sex, attribution, and age [8,9]. In this study, we extracted the incident number of esophageal cancer and gastric cancer cases and demographic data by sex and age groups in China from 1990 to 2019 to calculate the crude incidence rate (CR), and we calculated the age-standardized incidence rate (ASIR) using the age composition of Segi’s (1960) world standard population.

### 2.2. Statistical Analysis

A logarithmic linear model was built with the Joinpoint Regression Program (version 4.3.1.0; Surveillance Research Program, National Cancer Institute of US) to analyze the time trend of upper gastrointestinal cancer incidence over time. This model served to establish the piecewise regression based on the time characteristics of the disease distribution and divide the study period into different intervals by several combined points. Each interval was subjected to trend fitting and optimization to evaluate the specific disease change characteristics of different intervals within the global time range in more detail [10]. The annual percentage change (APC) and average annual percentage change (AAPC) with 95% confidence intervals (CIs) were calculated.

We evaluated the impact of the age, period, and cohort effects on upper gastrointestinal cancer incidence by building an age–period–cohort model. The age effect represents the onset risk of upper gastrointestinal cancer in different age groups, the period effect represents the impact of the time change on the onset risk of all age groups, and the cohort effect represents the change in the onset risk among different birth cohorts. The general logarithmic linear form of the age–period–cohort model is $\rho =\alpha_{a}+\beta_{p}+\gamma_{c}$, where *α* represents the age effect, *β* represents the period effect, and *γ* represents the cohort effect. According to Holford, if the age, period, and cohort trends are orthogonally decomposed into linear and non-linear parts, the model analysis should be performed according to the estimable function algorithm [11,12]. The model mainly includes the following indicators: (1) net drift is the overall logarithmic linear trend shown by the period and birth cohort, which may be used as the overall AAPC of the outcome indicator; (2) local drift is the logarithmic linear trend of each age group by period and birth cohort, which indicates the AAPC of the outcome indicator in each age group; (3) longitudinal age curve is the period drift-corrected rate specific to the longitudinal age of the reference cohort; (4) period *RR* is the relative risk of a period with respect to the reference period with the age and non-linear cohort effects corrected; (5) cohort *RR* is the relative risk of a cohort with respect to the reference cohort with the age and non-linear period effects corrected.

To evaluate the influence of the age, period, and cohort effects on the incidence of upper gastrointestinal cancer during the above study period, we used the age–period–cohort model online analysis tool provided by the National Institutes of Health [13]. In this study, we divided the incident number of upper gastrointestinal cancer cases and demographic data from 1990 to 2019 into an interval of 5 consecutive years; there were no incident cases among the population younger than 14 years old, so we divided the population into 15 age groups using an interval of 5 consecutive years, starting from the age of 15 (15–19, 20–24, …, 85+). All statistical tests were two-sided, and the difference was considered to be statistically significant when *p* < 0.05.

## 3. Results

### 3.1. Incidence of Upper Gastrointestinal Cancer in China from 1990 to 2019

From 1990 to 2019, there were 21,196,471 cases of upper gastrointestinal cancer in China, among which 14,934,040 were male cases and 6,262,431 were female cases. The CR of upper gastrointestinal cancer was 53.45/100,000, with 73.34/100,000 for males and 32.45/100,000 for females (male to female ratio: 2.26). The ASIR was 45.71/100,000, with 66.63/100,000 for males and 25.94/100,000 for males (male to female ratio: 2.57).

The incident number of upper gastrointestinal cancer cases in China was 491,022 in 1990 and 890,941 in 2019, an increase of 181.44%. The incident number of upper gastrointestinal cancer cases in males increased by 204.10%, from 323,000 in 1990 to 659,255 in 2019, and the incident number of cases in females increased by 137.89%, from 168,022 in 1990 to 231,686 in 2019.

### 3.2. Change in the Incidence of Upper Gastrointestinal Cancer in China from 1990 to 2019

**Figure 1:**
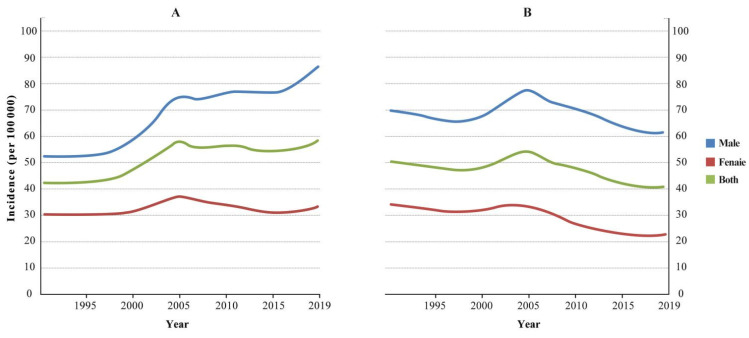
Incidence trends of upper gastrointestinal cancer in China from 1990 to 2019. (**A**) Crude incidence rate. (**B**) Age-standardized incidence rate.

The CR of upper gastrointestinal cancer in China increased by 51.01%, from 41.48/100,000 in 1990 to 62.64/100,000 in 2019. The CR of upper gastrointestinal cancer among males increased by 71.83%, from 52.93/100,000 in 1990 to 90.95/100,000 in 2019, and the CR of upper gastrointestinal cancer among females increased by 13.38%, from 29.30/100,000 in 1990 to 30.22/100,000 in 2019. The ASIR of upper gastrointestinal cancer decreased by 26.30%, from 50.77/100,000 in 1990 to 37.42/100,000 in 2019. The ASIR of upper gastrointestinal cancer among males decreased by 15.84%, from 68.95/100,000 in 1990 to 58.03/100,000 in 2019, and the ASIR of upper gastrointestinal cancer among females decreased by 45.28%, from 33.57/100,000 in 1990 to 18.37/100,000 in 2019 (Figure 1).

### 3.3. Time Trend of Upper Gastrointestinal Cancer Incidence in China from 1990 to 2019

The trends of the CR and ASIR of upper gastrointestinal cancer in China from 1990 to 2019 were divided into the following four periods: 1990–1998, 1998–2004, 2004–2016, and 2016–2019 (see Table 1 for the change trend in each period). The AAPCs of the CR and ASIR of upper gastrointestinal cancer in China from 1990 to 2019 were 1.42 (95% CI: 1.23 to 1.59%) and −1.12 (95% CI: −1.21 to −0.94), respectively, with values of 1.93 (95% CI: 1.71 to 2.12) and −0.56 (95% CI: −0.81 to −0.31) for males and 0.44 (95% CI: 0.25 to 0.65) and −2.07 (95% CI: −2.20 to −1.94) for females (Table 1).

### 3.4. Age–Period–Cohort Model Analysis of Upper Gastrointestinal Cancer

Using the age–period–cohort model, we analyzed the incidence of upper gastrointestinal cancer in China from 1990 to 2019 by sex. The Wald chi-square test showed statistical significance in the net drift, local drift, age deviation, period deviation, cohort deviation, period *RR,* and cohort *RR* of the male, female, and overall populations, with respect to the incidence of upper gastrointestinal cancer. The net drift of the incidence of upper gastrointestinal cancer was −0.83 (95% CI: −1.02 to −0.64), with −0.24 (95% CI: −0.39 to −0.08) for males and −2.25 (95% CI: −2.51 to −1.99) for females (Table 2).

**Figure 2:**
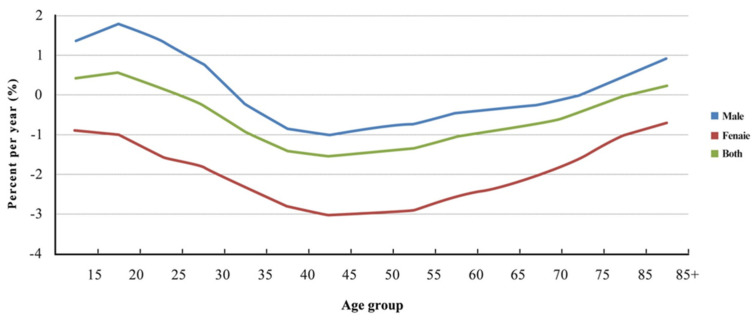
Local drifts in the incidence rate of upper gastrointestinal cancer in China from 1990 to 2019.

The local drifts of the 35–79 age groups of males and all age groups of females were less than 0, i.e., the incidences of upper gastrointestinal cancer in the above age groups decreased each year. The age groups of males from 40 to 59 years old showed a greater decrease, with the maximum decrease observed in the 45–49 age group (local drift = −1.01). A greater decrease in incidence among females was also observed in the age groups from 40 to 59 years old, with the 45–49 age group showing the maximum decrease (local drift = −3.03) (Figure 2).

**Figure 3:**
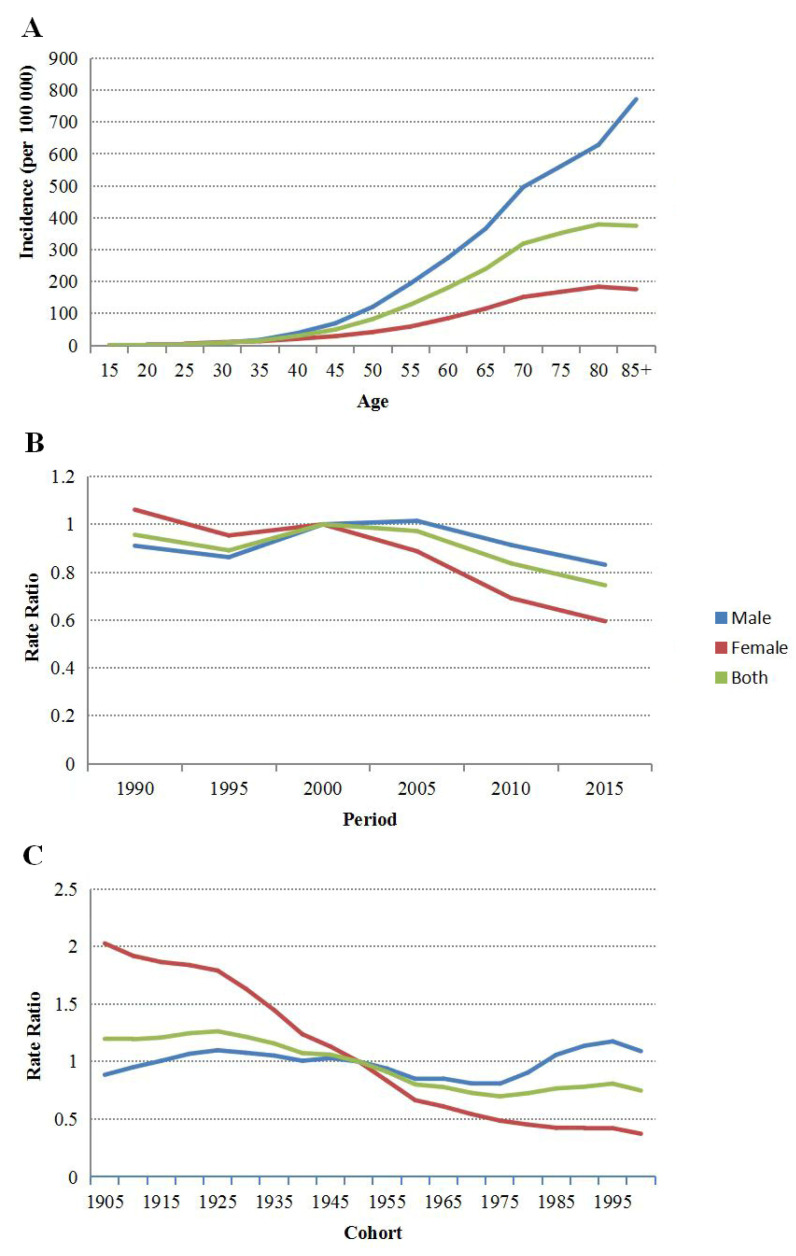
Age–period–cohort analysis of upper gastrointestinal cancer incidence in China from 1990 to 2019: (**A**) age effect; (**B**) period effect; (**C**) cohort effect.

For the age effect, with the cohort effect and period effect corrected, the onset risk of upper gastrointestinal cancer in the male, female, and overall populations gradually increased with age from the 15–19 age group to the 85+ age group. For the onset risk of males and females in the same age group, the onset risk of females aged 15–34 years was slightly higher than that of their male counterparts, while males showed a significantly higher onset risk than females in the 35+ age groups, especially in the high age groups. The upper gastrointestinal cancer onset risk in the 85+ age groups of males and females were 771.61/100,000 person/years and 175.94/100,000 person/years, respectively (Figure 3).

For the period effect, with the age and birth cohort effects adjusted and using 2000–2004 as the control group, the onset risk among males increased from 0.91 in 1990–1994 to 1.01 in 2005–2009 and then decreased gradually; the female and overall populations showed an onset risk trend that approximated that of males (Figure 3).

For the cohort effect, with the age and period effects corrected and using the 1946–1950 birth cohort as the control group, for males, the 1921–1925 birth cohort showed the maximum onset risk (1.10); the subsequent birth cohorts showed a gradually decreased onset risk until the 1971–1975 birth cohort (0.81), and the birth cohorts thereafter showed an increased onset risk again. For females, the birth cohort before 1905 showed the maximum onset risk (2.03), and the subsequent birth cohorts showed a monotonic onset risk decline trend. For the overall population, the 1921–1925 birth cohort showed the maximum onset risk (1.26), and the birth cohorts after 1926 showed a gradually decreased onset risk (Figure 3).

## 4. Discussion

In this study, we analyzed the data regarding upper gastrointestinal cancer incidence in China from 1990 to 2019 using Joinpoint software based on GBD2019 data. According to this study, the CR showed an upward trend, with AAPC being 1.42 (95% CI: 1.23 to 1.59), and the ASIR showed a downward trend, with AAPC being −1.12 (95% CI: −1.21 to −0.94). The GBD incidence data were estimated from multiple sources, such as previous literature reports, disease surveillance results, and health service statistics, using several methods, such as data fitting, filling, and correction [5]. A cancer registry data-based analysis also showed a downward trend of the standardized incidence of upper gastrointestinal cancer in China from 2000 to 2015, with AAPC being −3.5 (95% CI: −4.4 to −2.7) [14]. The overall cancer incidence increased slightly in China at the same time, in which the incidence of colorectal cancer, lung cancer and breast cancer increased faster [15]. The trend analyses based on the GBD estimated data and cancer registry data delivered the same conclusion that the CR of upper gastrointestinal cancer showed an upward trend, while the ASIR showed a downward trend. This situation is related to the significant change in the age composition of China’s population over the past 30 years. According to the data from the China National Bureau of Statistics, China’s elderly population aged older than 65 years accounted for 5.63% in 1990, 6.81% in 2000, 8.07% in 2010, and 14.2% in 2021 [16] and is expected to be nearly 30% in 2050 [17]. This indicates the trend of a decreasing number of young people and an increasing number of old people in China’s population. It is expected that China’s population growth will be stagnant in the future and that its degree of population aging will continue to increase [6].

There is a sex difference in the incidence of upper gastrointestinal cancer in China. The results of this study show that the CR among males is 2.26 times that among females, which is similar to the global situation [1]. From the age effect of the age–period–cohort model, it can be observed that males in the 35+ age groups were at a higher risk than females, especially in the high age groups. The onset risks of males and females decreased from 0.91 to 0.83 (decreased by 9.89%) and from 1.06 to 0.59 (decreased by 44.34%), respectively, with females showing a greater decrease. The sex difference in the incidence of upper gastrointestinal cancer is mainly caused by risk factors such as smoking, drinking, and unhealthy dietary behaviors, as well as differences between males and females in cancer prevention and treatment literacy. The smoking and drinking rates among males are 53.12% and 64.5%, respectively, which are far greater than those among females (3.02% and 23.1%) [18,19]. A meta study showed a synergistic effect and positive interaction of smoking and drinking on the occurrence of esophageal cancer, inducing the cancerization of normal esophageal tissues [20]. Moreover, female Chinese residents show higher cancer prevention and treatment literacy [21], as well as self-health awareness compared to male residents, leading to the reduction in some unhealthy behaviors. In addition, some studies show that female hormones (estrogen) can protect against gastric cancer [22].

The age effect is a major factor in the age–period–cohort model. Age is the most common risk factor for cancers. With age, the cumulative effect of carcinogenic factors will emerge, the body repair function will decrease, the risk of genetic mutation will increase, the function of the immune system will decrease; thus, the risk of cancer will increase significantly [23]. The backward trend of the average age of diagnosis may be related to the aging and prolonged average life expectancy of the Chinese population. Due to the cumulative effect of risk factors, the elderly have a relatively longer cumulative exposure to the risk factors, and thus suffer a relatively higher onset risk. Therefore, the incidence in the high age group is still at a high level, ultimately leading to a backward trend in the average age of esophageal cancer and gastric cancer diagnosis. In this study, from the local drift of the incidence of upper gastrointestinal cancer in all age groups, a decrease in the incidence was observed more often in the 40–59 age groups of both males and females. Therefore, in conclusion, the upper gastrointestinal cancer prevention window should be moved backward, and it is necessary to strengthen prevention and control among people aged 45 to 74 years [24].

The period effect of the incidence of upper gastrointestinal cancer showed a downward trend, which was related to China’s formulation of a series of cancer prevention and treatment plans and the focus on the reinforcement of primary and secondary preventive measures. Since the 1990s, realizing the severe situation of cancer prevalence, the Chinese government has issued various guiding documents at different stages of cancer prevention and control [25,26]. In these important documents, health policies must be implemented to combine cancer control with the prevention and treatment of other diseases. They set the objectives for cancer prevention and control, define the priority cancers to be prevented and controlled (including upper gastrointestinal cancer), and emphasize the leading role of the government. The five-year survival rate of cancer patients in China gradually increased from 30.9% in 2003–2005 to 40.5% in 2015, and the smoking prevalence among males in China has decreased by 1% every year, which indicates that China’s cancer control plan has achieved its preliminary effect [27,28]. With respect to the prevention and treatment of upper gastrointestinal cancer, various primary preventive measures, such as drinking water and toilet improvement, mold prevention and amine removal, nutritional supplementation, and changing bad living habits, have been implemented in the high-incidence regions, achieving favorable effects, as shown by a decrease in the cumulative mortality of gastric cancer from 4.28% to 3.84% [29]. With respect to the secondary preventive measures, the Cancer Early Diagnosis and Early Treatment Program in Rural China, Cancer Early Diagnosis and Early Treatment Program in Huai River Basin, and Cancer Early Diagnosis and Early Treatment Program in Urban China were initiated successively from 2005 to 2012, all of which included upper gastrointestinal cancer in their screening [30]. Studies show that providing endoscopic screening for upper gastrointestinal cancer in the high-risk rural regions may reduce the incidence and mortality of esophageal cancer and gastric cancer [4,31,32]. More than 2.16 million people have undergone endoscopic gastrointestinal examinations in 194 counties, with the detection rate being 2.05%, and the early diagnosis rate reaching 70% [33]. In such a large-scale screening, the individuals with precancerous lesions can be discovered, and timely interventions may be performed to prevent the occurrence of cancer and reduce the risk of the individuals developing upper gastrointestinal cancer. The period effect of the overall downward trend of the onset risk of upper gastrointestinal cancer benefits from the development of scientific and reasonable cancer prevention and control plans, as well as the effective implementation of primary and secondary preventive measures.

Based on the recognized risk factors for esophageal cancer and gastric cancer [5,34,35,36], the risk factors for upper gastrointestinal cancer mainly include *Helicobacter pylori* infection, smoking, drinking, overheated food and drinks, a high salt diet, moldy or pickled food, insufficient fruit and vegetable intake, deficiency of trace elements, etc. Intake of low-nutrient or nitrosamine-contaminated food may be a key factor in the previously high prevalence of upper gastrointestinal cancer in China [37]. In the past 30 years, in addition to population aging and rapid economic development, China also experienced a significant improvement in its residents’ living standards, housing conditions and nutritional status, and improvements in the awareness of cancer prevention. Some scholars believe that the decrease in the incidence of esophageal cancer in China may be related to economic growth, improvement of dietary habits, and changes in food storage methods [34]. Since the 1980s, numerous Chinese residents have begun to use refrigerators to store their food, reducing the decay of food, and thus the intake of nitrosamine-contaminated substances. A meta-analysis showed that compared to families who began to use refrigerators later, families using refrigerators earlier were at a lower risk of gastric cancer [38]. Chinese residents’ average daily salt intake has significantly decreased from 12.9 g in 1997 to 8.4 g in 2011 [39]. Moreover, the increase in household income promotes an increase in the intake of vegetables and fruits. In 1991, the daily per capita consumption of vegetables and fruits was 325.5 g, which increased to 352.7 g in 2002 and 452.95 g in 2013 [40,41]. The decrease in the incidence of gastric cancer was even called an “unexpected victory” in gastric cancer prevention, which can be attributed to the decrease in the consumption of salt and the storage of food in refrigerators or freezers. This change in storage methods led to an increase in residents’ consumption of fruits and fresh vegetables [36,42]. Chronic *Helicobacter pylori* infection is the highest risk factor for gastric cancer. The infection rate of *Helicobacter pylori* in China significantly decreased from 58.3% in 1983–1994 to 40.0% in 2015–2019 [43], and the decrease in the infection rate was related to the improvement of sanitary conditions, living quality, and the economic situation of families. A comparison of the results of several national epidemiological surveys on smoking from 1991 to 2011 indicated that the smoking rate decreased from 60.6% to 51.6% among males and from 4.0% to 2.9% among females, indicating a downward trend of smoking among residents [44]. These factors may be contributors to the reduced onset risk of upper gastrointestinal cancer among the birth cohorts born after 1926. Meanwhile, the dietary pattern of Chinese residents has been gradually westernized, and Chinese residents eat more processed and fatty meat. However, high unsaturated fat diets could increase the probability of occurrence of the gastric cancer [45].

It is worth noting that the disease spectrum of China has transitioned from the infectious disease-dominated spectrum to the chronic non-infectious disease (e.g., diabetes mellitus, cardiac-cerebral vascular diseases and cancers)-dominated spectrum [46]. The prevalence of diabetes mellitus and the number of deaths due to cardiovascular diseases has increased greatly [47,48]. In some populations, especially in the elderly population, the probability of death due to the cardiac-cerebral vascular diseases is higher, which might lead to a decrease in the probability that cancer patients are diagnosed, and to a decrease in the incidence of cancers, including upper gastrointestinal cancer.

### Strengths and Limitations

In this study, we analyzed the onset trend of upper gastrointestinal cancer using Joinpoint software and the age–period–cohort model. Joinpoint divides the long-term trend line of the disease into several segments and describes them. The short-term results are more accurate and widely used for the trend analysis of the incidence and mortality data of cancers. The advantage of the age–period–cohort model lies in its ability to analyze the trend of a disease by adjusting the age, period, and cohort synchronously. This model is mainly used for descriptive epidemiology and to analyze the incidence and mortality trends of chronic and infectious diseases. However, this study has some limitations. First, the data used in this study cannot be classified by province or city and rural region, so it is impossible to analyze the differences in the regional distribution of upper gastrointestinal cancer in China. Second, the data may be influenced by ecological fallacy, since an explanation of the results at the population level is not necessarily suitable for individuals and needs to be further validated in future individual-based studies.

## 5. Conclusions

China has developed cancer prevention and control plans in the past 30 years and has focused on the reinforcement of primary and secondary preventive measures, leading to a downward trend in the standardized incidence of upper gastrointestinal cancer. However, it is still the second most common cancer and the fifth cause of years of life lost [4]. Population aging in China is becoming increasingly serious, and some cancer risk factors have not yet been effectively controlled [49], posing challenges to the prevention and control of upper gastrointestinal cancer in China. In 2016, the Chinese government issued the “Health China 2030” Planning Outline [50], which proposed the important core indicator of reducing “premature mortality of major chronic diseases”, i.e., to increase the 5-year survival rate by increasing the early diagnosis rate of cancer. Therefore, increasing residents’ core awareness of prevention and treatment, expanding screening among the high-risk population, and increasing the early diagnosis rate of screening will be important in the next step towards the prevention and control of upper gastrointestinal cancer in China.

## Figures and Tables

**Table 1:** Incidence trend of upper gastrointestinal cancer in China from 1990 to 2019.

Trend	Year	APC (95% CI)	*p*-Value	AAPC (95% CI)	*p*-Value
**CR**					
Male					
Trend I	1990–1998	0.68 (0.32, 1.04)	<0.05	1.93 (1.71, 2.12)	<0.05
Trend II	1998–2004	6.96 (6.28, 7.64)	<0.05		
Trend III	2004–2017	0.22 (0.07, 0.37)	<0.05		
Trend IV	2017–2019	3.22 (0.79, 5.70)	<0.05		
Female					
Trend I	1990–1998	0.21 (−0.03, 0.44)	0.09	0.44 (0.25, 0.65)	<0.05
Trend II	1998–2004	4.43 (3.97, 4.89)	<0.05		
Trend III	2004–2015	−2.31 (−2.45, −2.16)	<0.05		
Trend IV	2015–2019	2.58 (1.96, 3.21)	<0.05		
Both					
Trend I	1990–1998	0.52 (0.25, 0.80)	<0.05	1.42 (1.23, 1.59)	<0.05
Trend II	1998–2004	6.07 (5.54, 6.60)	<0.05		
Trend III	2004–2016	−0.49 (−0.63, −0.36)	<0.05		
Trend IV	2016–2019	2.66 (1.65, 3.68)	<0.05		
**ASIR**					
Male					
Trend I	1990–1998	−1.17 (−1.55, −0.78)	<0.05	−0.56 (−0.81, −0.31)	<0.05
Trend II	1998–2004	4.11 (3.40, 4.82)	<0.05		
Trend III	2004–2017	−2.44 (−2.59, −2.28)	<0.05		
Trend IV	2017–2019	0.15 (−2.34, 2.69)	0.90		
Female					
Trend I	1990–1998	−1.71 (−1.95, −1.47)	<0.05	−2.07 (−2.20, −1.94)	<0.05
Trend II	1998–2004	2.09 (1.63, 2.56)	<0.05		
Trend III	2004–2015	−5.14 (−5.28, −4.99)	<0.05		
Trend IV	2015–2019	−0.49 (−1.12, 0.16)	0.13		
Both					
Trend I	1990–1998	−1.32 (−1.60, −1.03)	<0.05	−1.12 (−1.21, −0.94)	<0.05
Trend II	1998–2004	3.48 (2.94, 4.02)	<0.05		
Trend III	2004–2016	−3.30 (−3.40, −3.11)	<0.05		
Trend IV	2016–2019	−0.33 (−1.34, 0.70)	0.51		

NOTE: APC, annual percentage change; AAPC, average annual percentage change; CI, confidence interval; CR, crude incidence rate; ASIR, age-standardized incidence rate.

**Table 2:** Age–period–cohort Wald chi-squared test of upper gastrointestinal cancer incidence in China from 1990 to 2019.

Null Hypothesis	Male			Female			Both		
	χ^2^	*df*	*p*-Value	χ^2^	*df*	*p*-Value	χ^2^	*df*	*p*-Value
Net drift = 0	4.12	1	<0.05	282.43	1	<0.05	76.92	1	<0.05
All age deviations = 0	2156.5	13	<0.05	772.96	13	<0.05	3113.23	13	<0.05
All period deviations = 0	240.48	4	<0.05	164.66	4	<0.05	350.02	4	<0.05
All cohort deviations = 0	83.19	18	<0.05	109.23	18	<0.05	123.94	18	<0.05
All period RR = 1	251.47	5	<0.05	492.88	5	<0.05	474.64	5	<0.05
All cohort RR = 1	131.49	19	<0.05	1027.18	19	<0.05	511.54	19	<0.05
All local drifts = net drift	77.05	15	<0.05	99.69	15	<0.05	113.61	15	<0.05
Net drift (95% CI)	−0.21 (−0.39, −0.02)			−2.25 (−2.51, −1.99)			−0.83 (−1.02, −0.64)		

NOTE: CI, confidence interval; *df*, degrees of freedom.

## Data Availability

The datasets analyzed in the current study are available from the GHDx website (https://vizhub.healthdata.org/gbd-results/ accessed 15 April 2022). In addition, all estimations used to perform the analyses and generate the figures are available from the corresponding author.
